# Probing the Formation of Dark Interlayer Excitons via Ultrafast Photocurrent

**DOI:** 10.1021/acs.nanolett.3c01708

**Published:** 2023-10-03

**Authors:** Denis Yagodkin, Abhijeet Kumar, Elias Ankerhold, Johanna Richter, Kenji Watanabe, Takashi Taniguchi, Cornelius Gahl, Kirill I. Bolotin

**Affiliations:** † Department of Physics, Freie Universität Berlin, Arnimallee 14, Berlin 14195, Germany; ‡ Research Center for Functional Materials, National Institute for Materials Science, 1-1 Namiki, Tsukuba 305-0044, Japan; § International Center for Materials Nanoarchitectonics, National Institute for Materials Science, 1-1 Namiki, Tsukuba 305-0044, Japan

**Keywords:** interlayer dark exciton, transition metal dichalcogenides (TMDs), 2D semiconductor heterostructures, time-resolved photocurrent, interlayer dark exciton dynamics, time-resolved differential reflectivity

## Abstract

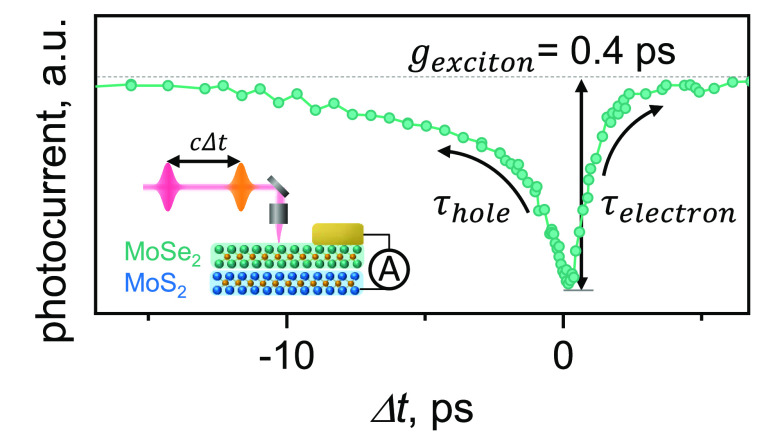

Optically dark excitons determine a wide range of properties of photoexcited semiconductors yet are hard to access via conventional time-resolved spectroscopies. Here, we develop a time-resolved ultrafast photocurrent technique (trPC) to probe the formation dynamics of optically dark excitons. The nonlinear nature of the trPC makes it particularly sensitive to the formation of excitons occurring at the femtosecond time scale after the excitation. As a proof of principle, we extract the interlayer exciton formation time of 0.4 ps at 160 μJ/cm^2^ fluence in a MoS_2_/MoSe_2_ heterostructure and show that this time decreases with fluence. In addition, our approach provides access to the dynamics of carriers and their interlayer transport. Overall, our work establishes trPC as a technique to study dark excitons in various systems that are hard to probe by other approaches.

Coulomb-bound electron–hole pairs (excitons) dominate the optical response of low-dimensional (0D, 1D, 2D) semiconductors.^1^ While early studies focused on optically allowed bright excitons, optically forbidden “dark” excitons have been studied much less. The radiative recombination of these latter excitons is suppressed as they involve states with nonzero total momentum, noninteger total spin, or spatially separated electron and hole wave functions.^1,2^ Due to the weak interaction with light, these states exhibit a long lifetime. This behavior of spin dark excitons is critical in understanding the efficiency limitations of charge collection in perovskite solar cells and photoluminescence (PL) in quantum dots.^3,4^ Momentum dark excitons are the lowest energy excitation in many transition-metal dichalcogenides (TMDs).^5^ Because of that, dark states likely dominate the long-range transport of excitons,^6,7^ determine temperature-dependent optical spectra,^8^ and are responsible for long-lived spin signals.^9,10^ Furthermore, dark excitons are promising for realizing interacting bosonic many-body states including the Bose–Einstein condensate and excitonic Mott insulator in TMDs.^11−13^ Special approaches are required to investigate the properties of the dark states due to their weak interaction with light. Spin dark excitons are brightened in a strong magnetic field,;^14^ however, the dynamics of the state is changed upon brightening. Conversely, the brightening of momentum dark excitons is challenging, thereby limiting the range of available techniques. For example, time- and angle-resolved photoemission spectroscopy (trARPES) or spectroscopies in the terahertz and far-infrared frequency ranges have been used to study dark excitons in TMDs and their heterostructures (HS).^15−18^ These approaches typically require large (hundreds of μm^2^ area) homogeneous samples or are performed at room temperature. Another approach to probe dark excitons, time-resolved photoluminescence (trPL),^12,19^ has a submicrometer spatial resolution but features a lower time resolution and does not work for states with vanishingly small oscillator strengths. As a result, many questions related to dark exciton formation, e.g., its time scale or the influence of phonon scattering and electron screening, remain unresolved.

Time-resolved photocurrent spectroscopy (trPC) has recently emerged as an approach to study optical processes in (2D) semiconductors.^20−23^ In trPC, a current across the sample is recorded vs the time delay between two light pulses impinging onto it. Critically, the technique is inherently sensitive to nonlinear processes. The approach applies to devices down to the nanometer scale and is compatible with other probes such as magnetic or electric fields, temperature, or strain. Here, we use the nonlinear response of two-color TrPC to probe the formation dynamics of dark excitonic species. To test our approach, we interrogate the formation dynamics of the commonly studied dark excitons in TMDs: interlayer excitons in MoS_2_/MoSe_2_ heterostructures.

## Toy Model of Time-Resolved Photocurrent

Our first goal of this work is to show that the dynamics of dark excitons, which are not accessible via conventional optical techniques, can be obtained from the time-dependent populations of free carriers. To understand this, we consider a minimal model of an optically excited semiconductor while analyzing the limitations of this model later on. We track the time-dependent densities of free electrons *N*_e_(*t*) and free holes *N*_h_(*t*). We assume that electron and hole populations can be excited together (direct excitation) or separately (indirect excitation), which we model by the generation functions *G*_e_(*t*) and *G*_h_(*t*). The relaxation processes of excited carriers can be broadly divided into two groups: linear (∼*N*_e/h_) and nonlinear (∼*N*_e/h_^2^, ∼*N*_e_*N*_h_ with carrier density. We focus on the coupled relaxation ∼*N*_e_*N*_h_, which describes the binding of an electron and a hole into an exciton^24^ (see Supplementary Note 1 for discussion on other parameters and detailed analysis of Auger-type and higher-order contributions). Overall, the carrier populations are described within our toy model by following rate equations
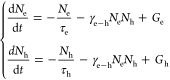
1Here τ_e/h_ is the linear relaxation times of electron and hole populations, and γ_e–h_ is the nonlinear exciton formation rate. Since the decay of excitons is several orders of magnitude slower compared to the relaxation/trapping of free electrons and holes,^25,26^ the density of the excitons is given by
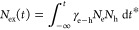


While the excitons described by *N*_ex_(*t*) can be dark (and hence hard to probe), their density can be reconstructed if we have experimental access to *N*_e_(*t*) and *N*_h_(*t*). To accomplish this, we numerically solve the above equations (see the Supporting Information for details). This simple model broadly characterizes systems with long-lived excitons.

**Figure 1:**
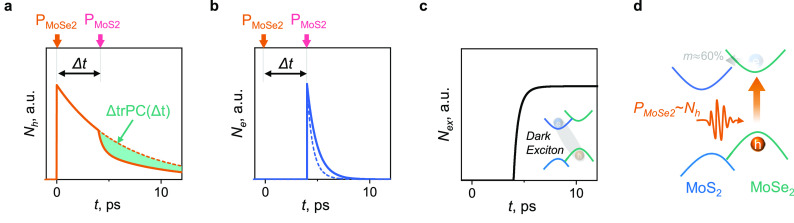
Excitation dynamics and photocurrent. Dynamics of holes (a), electrons (b), and excitons (c) modeled by eq 1. Solid and dashed lines correspond to nonzero and zero exciton formation rate (γ_e–h_). After the excitation by a pulse resonant with the MoSe_2_ band gap (*P*_MoSe_2__) at *t* = 0, the hole population (*N*_h_) decays exponentially with the rate τ_h_. This decay is accelerated after electrons are excited (*P*_MoS_2__) at Δ*t* ≈ τ_h_ = 4 ps in the case of γ_e–h_ ≠ 0. The quantitative measure of this acceleration, the shaded area in (a) is on the one hand determined by γ_e–h_ and, on the other hand, can be detected in a time-resolved photocurrent (trPC) experiment. (d) An optical pump pulse in resonance with MoSe_2_ bandgap (*P*_MoS_2__) excites predominantly holes in the VBM of the heterostructure, while a MoS_2_ resonant pulse excites electrons in CBM (not shown). Binding of electrons and holes in individual layers yields dark interlayer excitons (inset in (c)).

To gain insights into behaviors that eq 1 describes, we employ a series of simplifying assumptions. We first assume that holes and electrons can be excited separately (the numerical solution is free of this assumption; dynamics in this case is shown in Figure S1). When only holes are excited at *t*_1_ = 0 ps and only electrons at, for example, *t*_2_ ≈ τ_h_ = 4 ps, the solution yields the dynamics shown in Figure 1a–c. Initially, the excited population of holes decays exponentially. After the second pulse arrives, electrons are generated (Figure 1b). The relaxation of holes speeds up due to the formation of excitons if γ_e–h_ is nonzero. Interestingly, we see that the population of excitons (Figure 1c) qualitatively follows the difference between the hole populations with zero and nonzero γ_e–h_ (dashed and solid lines in Figure 1a). For the noninteracting case (γ_e–h_ = 0), the hole density is not affected by the second pulse exciting electrons (as in a single pulse excitation case), and therefore the population of excitons can also be equated to the difference of hole densities between a single pulse excitation (*G*_h_ ≠ 0; *G*_e_ = 0) vs two pulse excitation (*G*_h_ ≠ 0; *G*_e_ ≠ 0). We see that, in principle, the dynamics of dark excitons can be obtained from the dynamics of free carriers.

An obvious challenge arises when applying this toy model to a realistic physical system. Conventional optical techniques, such as transient reflectivity spectroscopy, detect combined contributions from photoexcited electron (*N*_e_), hole (*N*_h_), and exciton (*N*_ex_) populations. Therefore, an alternative technique sensitive to charge carriers is needed. To address this problem, we use time-resolved photocurrent spectroscopy as our measurement technique. Generally, photocurrent spectroscopies have the advantage of being directly sensitive to photogenerated electrons/holes while being insensitive to (neutral) excitons.^21,22^ In trPC, the system is illuminated by two optical pulses separated by time interval Δ*t*: *G*_h_(*t*_0_) and *G*_e_(*t*_0_ + Δ*t*), which generate populations of *N*_h_^0^ and *N*_e_^0^, respectively. The DC current across the material is recorded. The trPC signal is defined as the difference in current with both pulses being present versus only a single pulse. In general, photocurrent is proportional to the total amount of free carriers generated in a system over time. For the systems under study, TMDs, the direct contribution of electrons to the photocurrent can be neglected to simplify analytical derivation, as their lifetime is much lower and contact resistance is higher than that of holes^21,27^ (see Supplementary Note 1). In that case, the photocurrent produced by only the first pulse is given by the area under the dashed orange curve in Figure 1a. The relaxation of the hole population, in this case, depends only on τ_h_ as *G*_e_ = 0 for a single pulse excitation. The same holds for γ_e–h_ = 0 since the nonlinear term in eq 1 vanishes for both cases. The photocurrent produced by two pulses is given by the area under the solid curve in Figure 1a and is smaller than that of a single pulse (dashed curve) because of holes recombining with photoexcited electrons. It can be shown analytically (Supplementary Note 4) that in the limit of small γ_e–h_ and τ_h_ ≫ τ_e_, the trPC scales linearly with the exciton formation rate
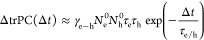
2

Here the relaxation time (denominator in the exponent) is τ_h_ if holes are excited first (positive delay time Δ*t* > 0) and τ_e_ if electrons are excited first (negative delay time Δ*t* < 0). This asymmetry stems from unequal generation terms *G*_e_ and *G*_h_ and allows the extraction of τ_e_ and τ_h_ as well as the rate of exciton formation from the single measurement.

We use the 2D heterostructure MoS_2_/MoSe_2_ as a system, where the generation rates for electrons and holes can be controlled separately. Indeed, the conduction band minimum (CBM) and valence band maximum (VBM) of the heterostructure reside in different materials, MoS_2_ and MoSe_2_, respectively (Figure 1d). Because of that, an optical pulse in resonance with, e.g., MoSe_2_ band gap (*P*_MoSe_2__) excites holes in the VBM of the structure (MoSe_2_), while the excited electrons can relax to the CBM (MoS_2_) through tunneling. Crucially, only around *m* ≈ 60% of these electrons reach the CBM of the structure in MoS_2_^28^ (Figure 1d). The remaining electrons are trapped and do not contribute to the photocurrent.^29,30^ These electrons are not affected by the second pulse.^31^ Similarly, a pulse resonant with the MoS_2_ band gap (*P*_MoS_2__) excites electrons to the CBM while *m* ≈ 60% of photoexcited holes reach the VBM of the structure (dynamics of carriers for this case is shown in Figure S1). Overall, we see that if *m* < 100%, the excitation of MoSe_2_ produces predominantly free holes in the VBM of the heterostructure, while the excitation of MoS_2_ produces predominantly free electrons in the CBM of the heterostructure.

We now apply eq 1 to model the excitation dynamics of the MoS_2_/MoSe_2_ heterostructure. The parameter *N*_e_(*t*) describes the electron density in the CBM of the structure (MoS_2_) and *N*_h_(*t*) describes the hole density in the VBM (MoSe_2_). The free electron/hole relaxation time (the term linear with *N*_e/h_) describes the combined contributions of defect capture,^32^ intervalley scattering,^33^ and radiative decay processes.^29,30^ The rate γ_e–h_ describes the formation of (dark) interlayer excitons. Of course, intralayer exciton are formed by optical pulses, especially in resonant excitation. However, optically excited intralayer excitons decay much faster (within <50 fs^15^) via charge separation across the heterostructure, compared to intralayer exciton recombination and electron/hole population cooling rate (>1 ps).^34−36^ Therefore, it is absorbed in the generation functions *G*_e_ and *G*_h_. The latter contain contributions from both pulses (i.e., *G*_e_(*t*) = *P*_MoS_2__(*t*,*t*_0_) + *m*·*P*_MoSe_2__(*t*,*t*_0_+Δ*t*); see the effect of *m* on the dynamics of charge carriers in Figure S1). Finally, the excitonic ground state of MoS_2_/MoSe_2_ is an interlayer exciton composed of an electron in MoS_2_ bound to a hole in MoSe_2_ (inset in Figure 1c). When the twist angle between the heterostructure layers (θ) is nonzero, the interlayer exciton has large in-plane momentum: , where *a* is the averaged lattice constant of the heterostructure.^37,38^ In this case, the radiative recombination must involve a phonon and the state is dark.^38^ Thus, the interlayer exciton decay (>100 ps) can be neglected on the time scales of the population buildup.^39^ Our next goal is to obtain the dynamics of *N*_ex_(*t*) via trPC.

## Time-Resolved Photocurrent

**Figure 2:**
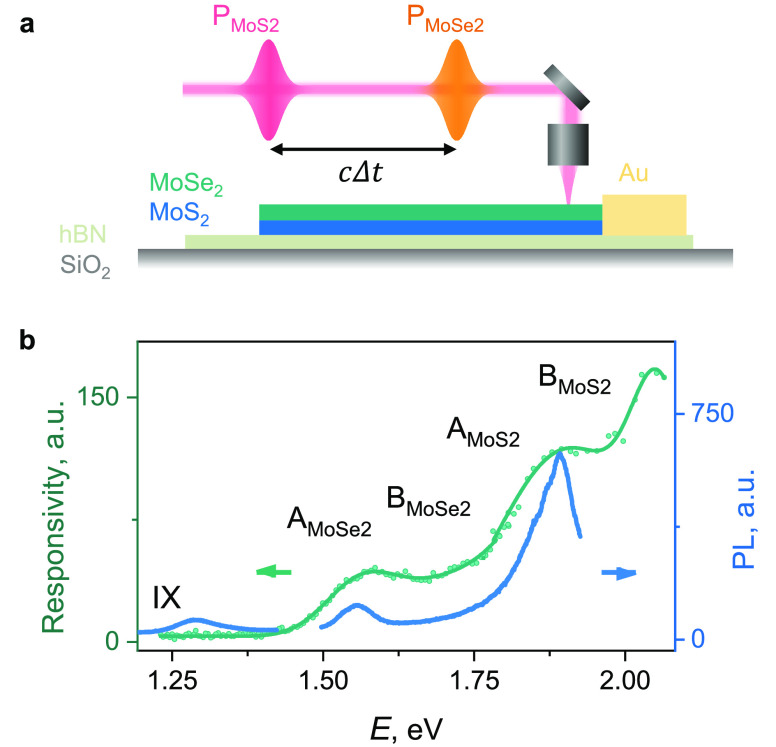
Sample structure and measurement techniques. (a) Scheme of two-color time-resolved photocurrent measurements (trPC). A photocurrent excited in the TMD heterostructure is measured vs time delay between a pulse in resonance with MoSe_2_ (*P*_MoSe_2__) and a pulse in resonance with MoS_2_ (*P*_MoS_2__). (b) Photocurrent responsivity (green dots, left axis) and PL (blue line, right axis) spectra of the MoS_2_/MoSe_2_ heterostructure. Intralayer A and B excitons of MoS_2_ and MoSe_2_ are seen (solid green fit) in PC at a bias voltage of 3.0 V. In PL an additional feature, an interlayer exciton (IX), is observed.

For trPC measurements, we fabricate MoS_2_/MoSe_2_ on hBN samples (Figure 2a; see Supplementary Note 2 for details). We observe the characteristic intralayer A and B excitons for both materials in static photocurrent spectroscopy in the heterostructure region (green dots in Figure 2b and Figure S9). In addition, weak photoluminescence due to interlayer excitons (IX) is observed at 1.3 eV^40^ (blue line in Figure 2b). For time-resolved measurements, the sample is illuminated with two time-delayed (with ∼10 fs precision) optical pulses, one in resonance with the MoS_2_ band gap and another with the MoSe_2_ band gap (*P*_MoSe_2__ = 50 μJ/cm^2^ unless otherwise stated). The photocurrent is measured with a lock-in amplifier synchronized to an optical chopper in one of the beam paths with no bias voltage applied. This measurement effectively allows us to evaluate the difference between single- and two-pulse responses, which corresponds to the area between the dashed and solid curves in Figure 1a.

**Figure 3:**
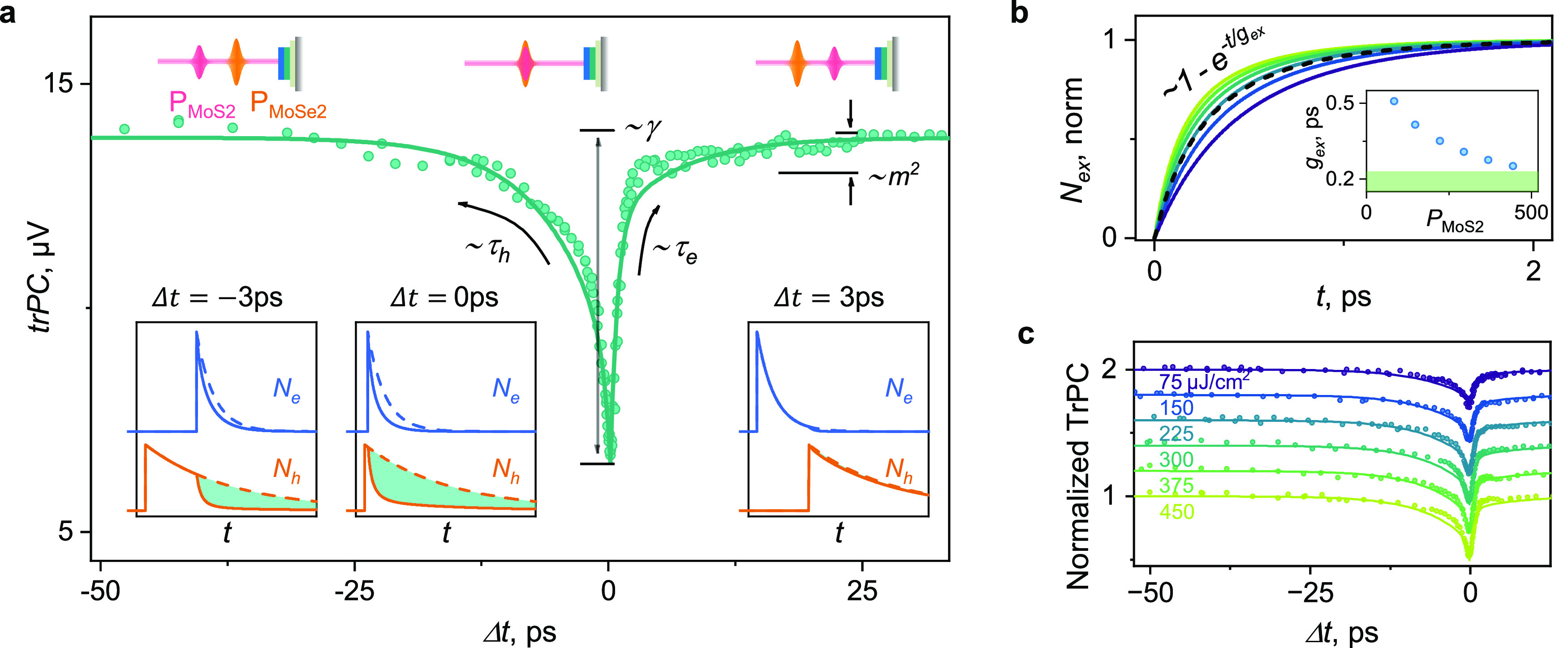
trPC measurements and extraction of exciton dynamics. (a) trPC response of a MoS_2_/MoSe_2_ heterostructure (points). The solid line shows simulated dynamics from the eq 1 with the following model parameters: τ_e_ = 1.0 ps, τ_h_ = 6.0 ps, interaction strength γ_e–h_ = 0.13 cm^2^/s, and electron/hole tunneling *m* = 55%. The insets display dynamics of holes (orange) and electrons (blue) for selected delays between pulses for the simplified case of *m* = 0. The difference between response to a single optical pulse (solid) and two pulses (dashed) is proportional to ΔtrPC (green area). (b) Simulated dynamics of interlayer exciton formation for data in (a) (black dashed line) and for other fluences *P*_MoS_2__ = 75, 150, 225, 300, 375, 450 μJ/cm^2^ (solid lines from purple to yellow). The exciton formation becomes faster for higher fluences, as quantified by fitting to , where *g*_ex_ is the exciton formation time. Inset: extracted *g*_ex_ for the aforementioned fluence range (blue points). The green area corresponds to the cross-correlation of the pulses. The formation time drastically decreases for high laser fluences. (c) Normalized fluence dependence of trPC response (points, each data set is offset by 0.2) and independent from measurement simulations using eq 1 with parameters from (a) (lines). At higher fluences (yellow), the trPC drop at zero time delay increases, suggesting a faster formation and higher number of excitons.

Figure 3a shows experimental trPC data (green dots) of the MoS_2_/MoSe_2_ sample vs delay Δ*t* between the excitation pulses. Positive delay corresponds to the pulses resonant with the MoS_2_ band gap arriving first. The most prominent features of the data are a strong dip at zero time delay and a pronounced asymmetry between positive and negative delays. We now show that these features can be understood within our toy model. First, a large drop in trPC suggests nonlinear interaction between the carrier populations produced by both pulses, described by γ_e–h_ in our model (eq 2). Second, the asymmetry can be understood from eq 2. It suggests that the lifetime of electrons photoexcited in MoS_2_ is much smaller than the lifetime of holes excited in MoSe_2_ (see insets in Figure 3a for the illustration of trPC at negative, zero, and positive time delays). Third, we note a slower decaying component at Δ*t* > 3 ps. This minor effect is missing in Figure 1a and occurs for *m* ≠ 0 (Supplementary Note 5). Its origin is the transfer of holes excited by P_MoS2_ from MoS_2_ to the VBM of the structure (MoSe_2_). The ratio between fast and slow decaying components is proportional to *m*^2^ (eq S12).

To obtain the precise values of the model parameters, we fit the numerical solution of eq 1 (the solid line in Figure 3a) to the experimental data. We obtain relaxation times of τ_h_ = 6.0 ± 0.5 ps, τ_e_ = 1.0 ± 0.2 ps, an interaction strength of γ_e–h_ = 0.13 ± 0.04 cm^2^/s, and a transfer efficiency of *m* = 55 ± 5%. The effect of the variation in every parameter is shown in Figure S2. Using the extracted values, we plot the generation dynamics of interlayer excitons vs excitation fluence (black dashed line in Figure 3b). Since the rate at which excitons are formed is proportional to *N*_e_*N*_h_ within our model (eq 1), its acceleration is expected with a higher density of electrons/holes, as seen in the simulations for several fluence values (solid lines in Figure 3b). We observe the formation time (inset of Figure 3b) dropping by more than a factor of 2 in the range of fluences between 100 and 500 μJ/cm^2^. Overall, the simulations suggest two key behaviors. First, we see a much faster relaxation of the electron population compared to holes. Second, we obtain a formation time of the exciton, *g*_ex_ = 0.4 ps, at our experimental incident fluence *P*_MoS_2__ = 160 μJ/cm^2^.

Next, we tested the predictions of these simulations. To check the dependence of exciton formation on fluence given by our model (Figure 3b), we carried out fluence-dependent trPC measurements. We keep the incident fluence of *P*_MoSe_2__ fixed, while *P*_MoS_2__ is varied in the range used in simulation: 75–450 μJ/cm^2^ (dots in Figure 3c). We see that the magnitude of the drop of the trPC at zero time delay increases with fluence from around 30% at 75 μJ/cm^2^ up to 50% at 450 μJ/cm^2^. The experimental data closely follow the independent predictions of the model (lines in Figure 3c), where we used the same parameters as in Figure 3a and only changed the fluence of the beam (*P*_MoS_2__). We extract the formation time of interlayer excitons *g*_ex_ (inset in Figure 3b) and find that it is more than halved in the given fluence range from 0.5 to 0.2 ps. We observe small deviations between simulated trPC and the experiment around the delay Δ*t* ≈ −5 ps and Δ*t* ≈ 2 ps at high excitation fluence (green in Figure 3c). The first deviation could be the signature of the contribution of higher-order terms in eq 1 (see Supplementary Note 1). The second deviation may be an effect of the electric field created by layer-separated carriers. This field reduces band offsets, resulting in a decrease of the interlayer transfer efficiency by Δ*m* ≈ 10% (Figure S3).

**Figure 4:**
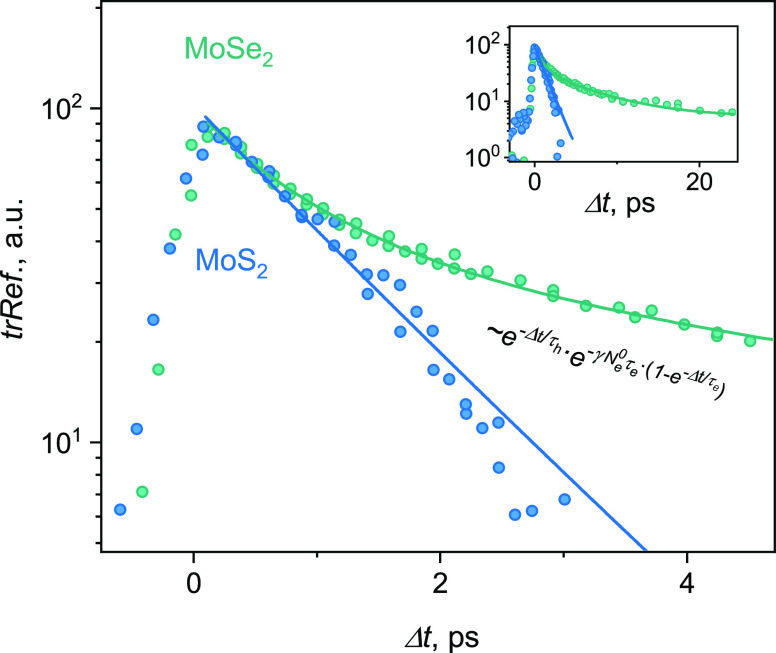
Testing the model: reflectivity dynamics. Semilog plot of time-resolved reflectivity signal from the MoSe_2_/MoS_2_ heterostructure. Green points correspond to the probe pulse in resonance with MoSe_2_ band gap (pump MoS_2_) and blue in resonance with MoS_2_ band gap (pump MoSe_2_). The inset shows a longer time scale. Lines are fits (eq S1) derived from the model in eq 1.

To independently check the dynamics of free carriers, we carry out two-color time-resolved reflectivity (trRef) measurements^26^ (Figure 4). In this approach, one optical pulse, e.g., in resonance with the MoSe_2_ band gap, excites both holes and electrons. Part of the electron population is transferred to the CBM in MoS_2_. The relaxation time of that electron population is probed by the second (probe) pulse in resonance with the MoS_2_ band gap, *P*_MoS_2__ = 150 μJ/cm^2^ (blue points in Figure 4). Conversely, the pump in resonance with MoSe_2_ band gap and the probe in resonance with MoS_2_ band gap detect the dynamics of holes in MoS_2_ (green points in Figure 4). Time constants for electron and hole populations obtained by fitting the data of Figure 4 to our model (Supplementary Note 3), τ_e_ = 1.4 ps and τ_h_ = 6.3 ps, are close to what is obtained from trPC, further validating that approach. In addition, the detailed analysis of this model suggests that the biexponential decay seen for holes (green in Figure 4) across a range of fluences (Figure S4), as well as observed in other works,^26,27^ originates from the formation of dark excitons.

## Conclusion and Outlook

To summarize the discussion above, we proposed a unique method to probe the electron–hole dynamics as well as the dynamics of dark excitons via time-resolved photocurrent spectroscopy. We extract the following parameters of the system: e–h coupling (γ_e–h_ = 0.13 ± 0.04 cm^2^/s), relaxation times of electrons and holes (τ_e_ = 1.0 ± 0.2 ps and τ_h_ = 6.0 ± 0.5 ps), and efficiency of interlayer transport (*m* = 55 ± 5%), for all fluence regimes. The interlayer exciton formation time varies from 0.2 to 0.5 ps in the range of fluences 450–75 μJ/cm^2^. It is useful to compare these values with those obtained by other approaches. Electron/hole lifetimes are consistent with those broadly reported from optical measurements.^9,29,36,41,42^ The discrepancy in the lifetimes of electrons and holes, with electrons exhibiting shorter lifetimes, likely arises from trapping at defect states located closer to the conduction band.^43−45^ The observed exciton formation time *g*_ex_ = 0.4 ps at 160 μJ/cm^2^ fluence matches the time scales reported in trARPES (∼230 fs),^15^ trTHz reflectivity (∼350 fs),^46^ and trFIR (∼800 fs)^47^ experiments. The interlayer transfer efficiency *m* has been estimated from THz measurements to be 50–70%,^28^ also close to the values here. Overall, our approach provides simple access to the dynamics of the dark excitons. Moreover, the proposed model describes trRef dynamics of heterostructures and explains the biexponential decay reported before.^26,27^

While our model of trPC based on eq 1 is transparent and matches the main observed behavior, its simplified nature necessitates several key approximations that may affect its validity. First, the model assumes that the entire photocurrent is produced by free electrons and holes quasi-instantaneously created by the excitation pulses. In reality, the photocurrent is also produced by nonradiative Auger processes^21,29^ and field-induced dissociation of both inter- and intralayer excitons.^21^ We believe that these effects are only relevant at high excitation fluences and large applied bias voltages (*V*_b_ > 5 V) as was shown before.^12,22^ Second, the model neglects the decay of the interlayer excitons. This is a well-controlled approximation given that their decay is 4 orders of magnitude slower compared to the rate of their formation.^25,26,39^ The dynamics of the intralayer exciton is absorbed within the model into the generation functions of the carriers. That is justified as long as the charge separation across the interface is much faster compared to other decay channels.^15,24^ Third, the separation of electron and hole dynamics in the data such as Figure 3 is facilitated by the difference of electron/hole generation functions (*G*_h_ ≠ *G*_e_ in eq 1) giving rise to a pronounced asymmetry between positive and negative time delays. Nevertheless, the same time-resolved photocurrent technique can also be applied to systems without such an asymmetry, for example, single-layer TMDs or perovskites. In that case, relaxation constants for electrons and holes should be obtained via an independent measurement, such as optical reflectivity. Finally, the model makes a simplifying assumption of a 1 – *m* fraction of carriers staying in the layer where they were excited and the fraction *m* tunnels across the layers. While this assumption matches our data as well as previously reported experiments by others,^28^ it can only be confirmed via future detailed studies of localized and free carrier dynamics.

To conclude, we demonstrate an approach for studying the dark exciton formation dynamics. In the future, this approach can be used to study other systems where the excitonic ground state is optically dark, e.g., monolayer TMDs, organic films, and perovskites. Unlike other approaches, trPC is fully compatible with other optical techniques (trRef and PL shown here, Kerr and ellipticity spectroscopies, second-harmonic generation), has a spatial resolution of hundreds of nanometers, and works at cryogenic temperatures. It will be particularly interesting to use trPC to uncover the effects of many-body interactions (exciton Mott transition, localization at low temperature), electric field, and twist angle on the exciton formation time.

## Data Availability

The data that support the findings of this study are available from the corresponding author upon reasonable request.

## Supporting Information Available

Dynamics of the holes, electrons, and interlayer excitons for nonzero interlayer transfer, effect of the parameter variation of the simulated trPC and carrier dynamics, high fluence effects in trPC, fluence dependence of trRef, and discussion of the model, methods, two-color time-resolved reflectivity, analytical solution of the charge carrier and exciton dynamics, and resulting the photocurrent and dynamics in case of nonzero transfer efficiency (*m* ≠ 0) (PDF)
