# Supplementary material for: Probing the Formation of Dark Interlayer Excitons via Ultrafast Photocurrent
Source: Nano Lett. 2023 Oct 3;23(20):9212–8. doi: 10.1021/acs.nanolett.3c01708 (PMC10603811; doi:10.1021/acs.nanolett.3c01708)
Supplement: Supplementary file 1 — nl3c01708_si_001.pdf [file nl3c01708_si_001.pdf]

Supplementary information

# Probing the formation of dark interlayer excitons via ultrafast photocurrent.

Denis Yagodkin[1], Abhijeet Kumar[1], Elias Ankerhold[1], Johanna Richter[1], Kenji Watanabe[2], Takashi Taniguchi[3], Cornelius Gahl[1], and Kirill I. Bolotin[1]

**Fig S1. Dynamics of the holes, electrons and interlayer excitons for non-zero interlayer transfer.**

**Fig S2. Effect of the parameter variation of the simulated trPC and carrier dynamics.**

**Fig S3 High fluence effects in trPC.**

**Fig S4 Fluence dependence of trRef.**

**Supplementary Note 1: Discussion of the model.**

**Supplementary Note 2: Methods.**

**Supplementary Note 3: Two-color time-resolved reflectivity.**

**Supplementary Note 4: Analytical solution of the charge carriers and excitons dynamics and resulting photocurrent.**

**Supplementary Note 5 Dynamics in case of non-zero transfer efficiency ($m \neq 0$).**

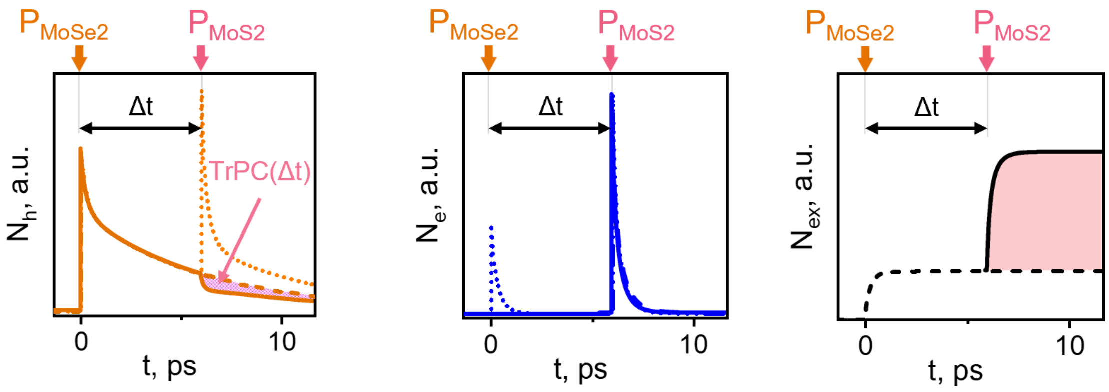


**Fig S1. Dynamics of the holes (orange), electrons (blue) and interlayer excitons (black) for non-zero interlayer transfer.** The dynamics of free carriers and excitons for case of non-zero $m$ (i.e., both optical pulses excite electrons and holes). Specifically, we assume that $G_e = N_e^0 + N_e^{\mathrm{MoSe2}} m = N_e^0 + N_h^0 m$, where $N_e^{\mathrm{MoSe2}}$ is density of electrons excited in $MoSe_2$ (equal to density of excited holes in $MoSe_2$), around $m \approx 60\%$ of which is transferred to CBM in $MoS_2$, $N_{e/h}^0$ is density of electrons (holes) excited in $MoS_2$ ($MoSe_2$). Similar for holes: $G_h = N_h^0 + N_e^0 m$. Orange lines correspond to time-dependent hole densities, blue – electron density and black – excitons density. Dotted lines represent the result of two pulse excitation, while dashed lines show single pulse response, $P_{MoSe2}$ for holes and $P_{MoS2}$ for electrons, respectively. Solid lines show difference between response to the two pulses (dotted line) and only second pulse, $P_{MoS2}$ for holes and $P_{MoSe2}$ for electrons (not shown). In the experiment, the solid line is measured as phase locked amplification filters out contribution to not-phase-locked second pulse. In case of interlayer excitons, dashed line shows interlayer excitons formed after first pulse excitation and regardless of the second pulse presence, hence similarly regarded as background. Similar to Fig. 1 of the main text, difference between two pulses (solid line) and single pulse (dashed line) is proportional to $\gamma_{e-h}$ and corresponding pink area is measured in trPC.

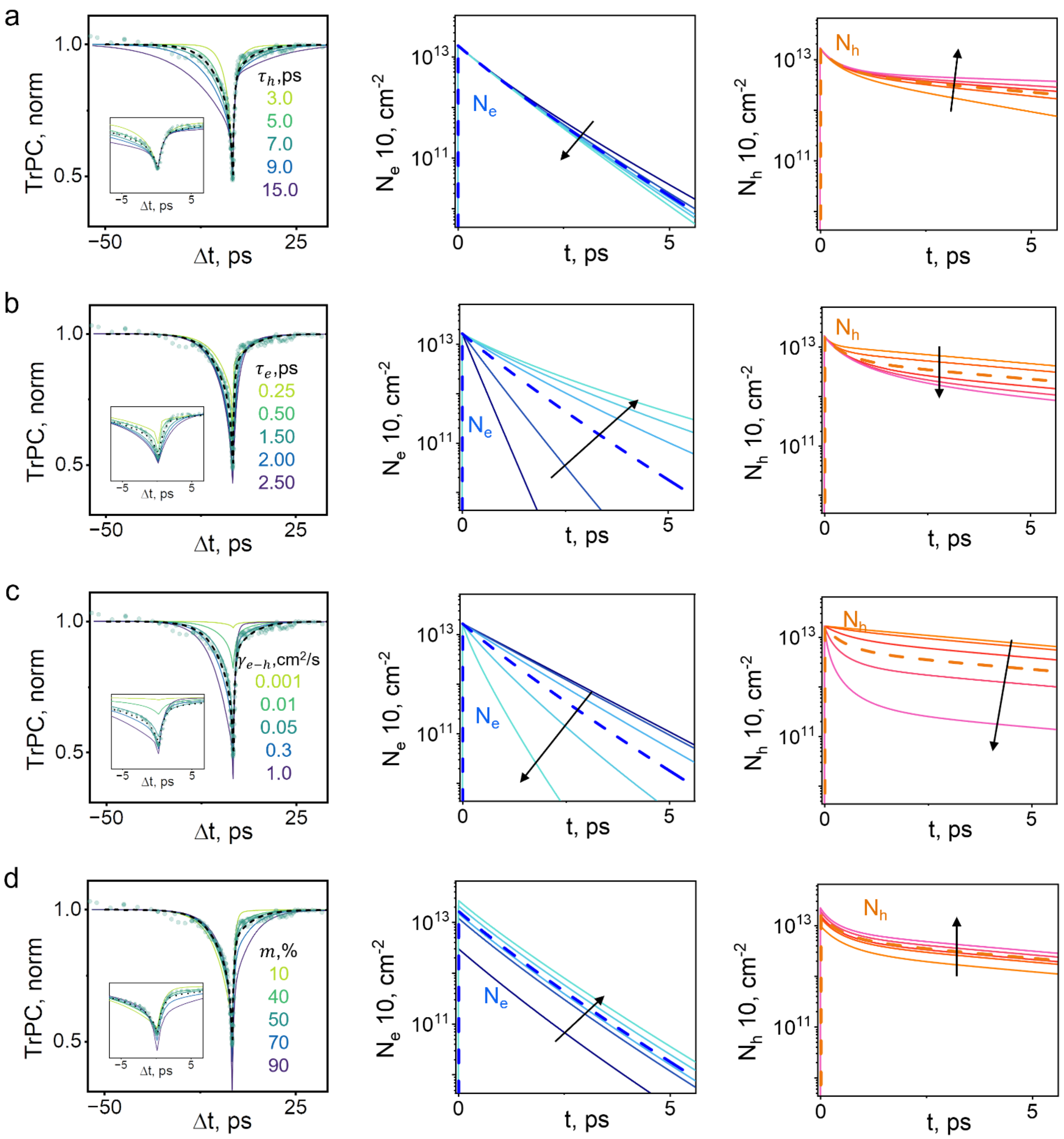


**Fig S2. Effect of the parameter ($\tau_h, \tau_e, \gamma_{e-h}, . m$) variation of the simulated trPC and carrier dynamics**. In a-d) we plot the result of the simulations when we vary the hole lifetime ($\tau_h$), election lifetime ($\tau_e$), electron/hole interaction strength ($\gamma_{e-h}$), and tunneling efficiency ($m$) respectively. In each case, left panels shows simulated trPC, middle and right panels show simulated dynamics of electrons and holes, respectively. We always use the simulation parameters used in the main text, $\tau_e = 1$ ps, $\tau_h = 6$ ps, interaction strength $\gamma_{e-h} = 0.13$ cm$^2$/s, electron/hole tunneling $m = 55\%$ as a baseline (dashed line in all the plots) and vary one parameter (solid lines from yellow to purple in trPC, blue to cyan in $N_e$ dynamics and orange to pink in $N_h$. Arrows point toward increase of the varied parameter)

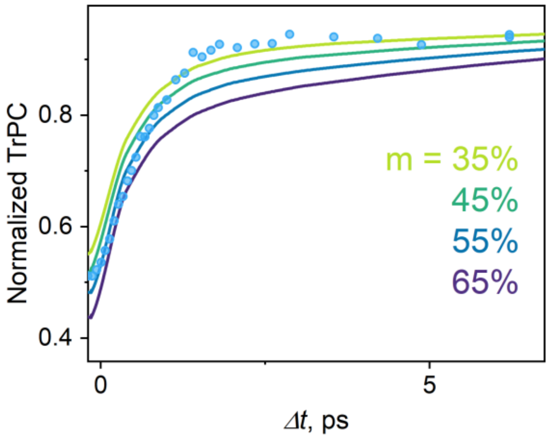


**Fig S3 High fluence effects in trPC.** Zoomed-in trPC response of the heterostructure (Fig. 3c) at high excitation fluence, 450 μJ/cm$^2$, blue dots and simulations (solid lines) run with interlayer transfer efficiency $m$ in range from 35% to 65% (green to purple). At higher fluence, field created by interlayer excitons and screening by carriers transferred across the interface reduce offset between $MoSe_2$ and $MoS_2$ bands. This leads to decrease of the charge separation efficiency at the interface, which is captured in our model (Eq. 1 of the main text) by $m$ - interlayer transfer efficiency parameter. At low fluence the fit of the simulation to the experiment yields $m = 55\%$ (Fig. 3c), however at larger fluence better agreement is achieved with $m = 45\%$.

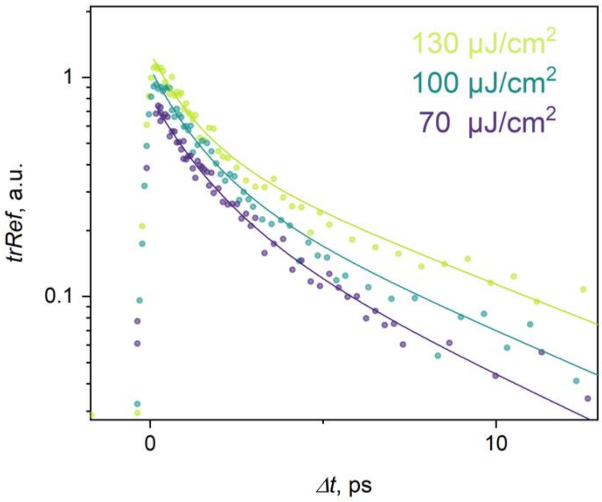


**Fig S4 Fluence dependance of trRef.** Semilog plot of time-resolved reflectivity signal from the $MoSe_2/MoS_2$ heterostructure. Fluence of the pump pulse in resonant with $MoS_2$ bandgap is varied between 70, 100, 130 μJ/cm$^2$ (purple to green dots) while fluence of probe pulse in resonance with $MoSe_2$ bandgap is kept constant. Lines are fits (Eq. S1) derived from the model in Eq.1 of the main text.

**Supplementary Note 1: Discussion of the model.**

While our model of trPC based on Eq. 1 of the main text is transparent and matches the main observed behaviors, it implies several key approximations that may affect its validity. In this note we discuss the following effects:

- Contribution of photoexcited electrons to photocurrent
- Auger-type processes
  - Auger decay of intralayer excitons
  - Auger decay of interlayer excitons
  - Auger deacy of charge carriers
- Higher order contributions to charge dynamics, e.g. trion formation

We dismiss electron contribution to photocurrent in the heterostructure. In general, photocurrent is proportional to the total amount of free carriers generated in a system over time, $a_e \int (-q) N_e dt + a_h \int qN_h dt$, where $a_{e,h}$ are extraction efficiencies for electrons and holes, and $q$ is the elementary charge[1]. For the systems under study, TMDs, $a_e \ll a_h$ and $\tau_e \ll \tau_h$, therefore the direct contribution of electrons to the photocurrent can be neglected [1,2] (Fig. S5).

In principle, Auger-type recombination could contribute to the processes we consider in our model. To analyze such contributions, distinguish Auger-type processes for three relevant species: intralayer excitons, interlayer excitons, and free carriers.

Auger decay of intralayer excitons results in an increase in the population of free carriers right after photoexcitation[3]. This effect competes with charge separation at the heterostructure interface (time constant below 50 fs) which also results in the conversion of intralayer excitons into free carriers. To estimate the Auger process for intralayer excitons, we use the exciton-exciton annihilation rate 4.3 $cm^2/s$ [3]. Considering the maximum fluence used in our study of 450 $uJ/cm^2$, we estimate the exciton-exciton annihilation time to be around $\tau_{ex-ex} \sim 10$ ps, three orders longer than the charge separation rate mentioned earlier. Zhu H, et al. study on heterostructures similarly shows no acceleration of decay at high fluence, contrasting with observations in monolayers[4]. Therefore, while Auger-type intralayer exciton-exciton annihilation is expected, it is negligible compared to the charge separation rate at the interface.

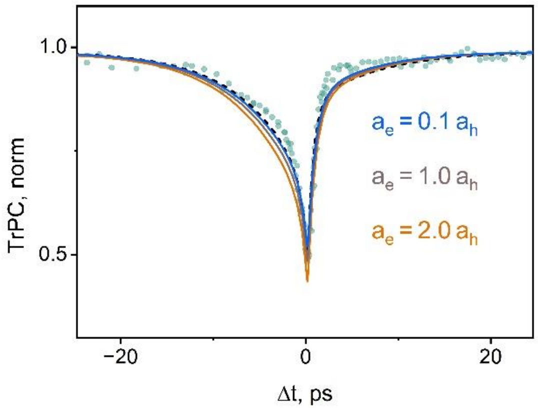


**Fig S5. The effect of electron extraction efficiency on trPC.** In the main text, we assumed that only holes contribute to the observed trPC signal due higher hole extraction efficiency ($a_h$) at the contact. Here, we consider the effect of non-zero electron extraction efficiency ($a_e$). We simulated trPC for $a_e = 0.1a_h$; $1.0a_h$; $2a_h$ (blue to orange respectively) using the approaches described in the main text. For comparison, the experimental trPC response of a $MoS_2/MoSe_2$ heterostructure (green points, same as main text Fig. 3) and the results of simulations for $a_e = 0$ (black dashed line, same parameters as in the main text) are also shown. We see that even if electron extraction efficiency were large, it would not significantly affect our simulated results. This is related to longer lifetime of holes compared to electrons (~6 ps vs. ~1 ps)

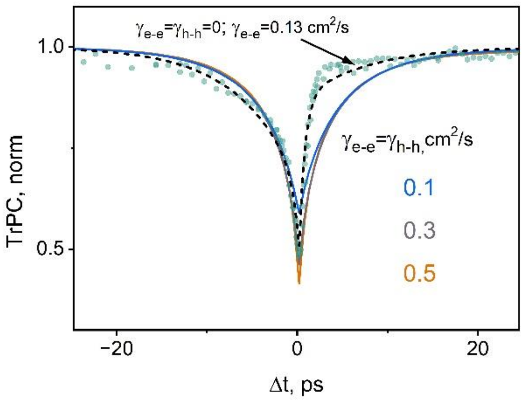


**Fig S6. The effect of hole-hole ($\gamma_{h-h}$) and electron-electron ($\gamma_{e-e}$) interactions on trPC**. In addition to the electron/hole interaction described in the Eq. 1 by the term $\gamma_{e-h}N_eN_h$, similar second order terms for electron-electron ($\gamma_{e-e}N_e^2$) and hole-hole ($\gamma_{h-h}N_h^2$) interactions may in principle be relevant. To understand potential contributions of these terms, we first considered the case when they are much larger compared to $\gamma_{e-h}$. Specifically, we simulated trPC described by Eq. 1 for $\gamma_{e-h} = 0$, $\gamma_{e-e} = \gamma_{h-h} =$ 0.1; 0.3; 0.5 $cm^2/s$ (solid curves, blue to orange respectively). For comparison, we plotted the observed trPC response of a $MoS_2/MoSe_2$ heterostructure (green points, also shown in Fig. 3 of the main text) and simulated dynamics used in the main text without e-e and h-h interactions $\gamma_{e-e} = \gamma_{h-h} = 0$, and $\gamma_{e-h}$ =0.13 $cm^2/s$ (the dashed black line). We see that for any values of $\gamma_{e-e}$ and $\gamma_{h-h}$ the simulated trPC is qualitatively different from what is observed experimentally – the trPC signal decays slower at positive decay time than at negative delay time. Moreover, by varying these parameters we found that the simulation can match experimental observations (fast decay for positive time and slow for negative time) only when electron-electron and hole-hole interaction at least order of magnitude smaller than electron-hole interaction. Therefore, we neglect $\gamma_{h-h}$ and $\gamma_{e-e}$ to simplify the model.

Interlayer excitons are expected to undergo Auger-type decay as well. However, the overall decay of interlayer excitons happens at a much longer timescale (~10 ns) than the one we investigate (~50 ps). Several studies report a shorter lifetime of interlayer excitons at increased fluence, but even that timescale is much longer than the timescale in our study[5–8]. This confirms that the Auger recombination of interlayer excitons doesn't affect our model.

Auger recombination of charge carriers is crucial to our model and competes with interlayer exciton formation. Analysis of time-resolved photocurrent (trPC) shows that the Auger process, even if present, is much less effective than interlayer exciton formation (Fig. S6). To confirm this observation, we conducted additional measurements of time-resolved reflectivity at low temperature (Fig. S7). In general, the free carrier lifetime increases at lower temperature compared to excitons, thereby facilitating Auger recombination. Measurements revealed a small, fast decaying component, which does not show significant dependence on fluence unlike expected for Auger: accelerating decay time and increasing relative amplitude. Several other studies have also observed the absence of Auger

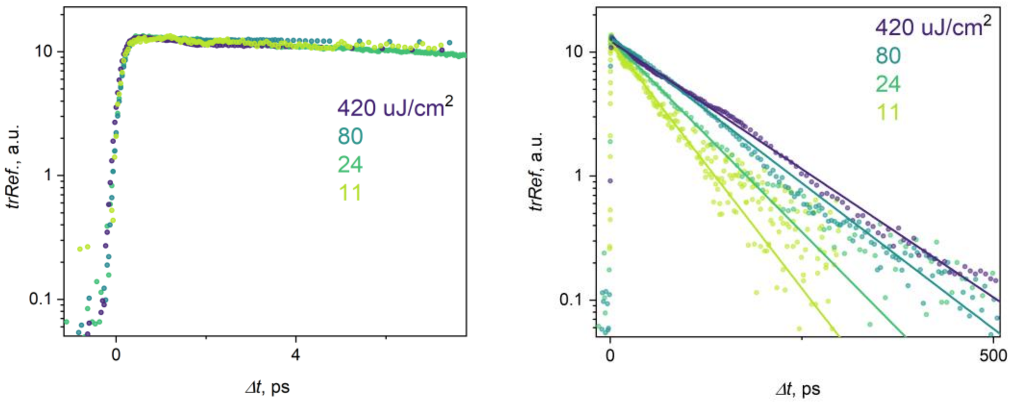


**Fig S7. Fluence dependence of trRef of a $MoSe_2/MoS_2$ heterostructure at low temperature.** Normalized time resolved reflectivity response measured with pump pulse resonant with the $MoSe_2$ bandgap (1.63 eV) and fluence in range between 11 and 420 $\mu J/cm^2$ (green to purple dots), while probe pulse resonant with $MoS_2$ bandgap (2.0 eV) and constant fluence of 33 $\mu J/cm^2$. Unlike expected for Auger process: sudden drop of the signal at short time scale (left panel) accelerating at higher fluence, we observe little variation in the wide range of fluence. At longer time scale (right panel) the signal decay even slows down at higher fluence. The slowdown could be due to defect state filling[10].

recombination in heterostructures, even at fluence higher than triggers Auger effects in monolayers[7,9]. The slowdown of the long-living component of reflectivity at high fluence may be due to defects filling up and increasing carrier lifetime[10].

We dismiss higher order terms corresponding to interlayer trions such as ~$\gamma_T N_e^2 N_h$ or ~$\gamma_T N_e N_h^2$ in Eq.1 of the main text, where $\gamma_T$ is nonlinear rate of trion generation. The simulation results including such terms are in Fig. S8. The parameter values yielding the best fit were found to be $\gamma_{e-h} = 0.085$ cm$^2$/s; $\gamma_T = 8{\cdot}10^{17}$ cm$^4$/s, which in relative terms stays below 10% of interlayer exciton formation rate ($\gamma_T \cdot \overline{N}_e \approx 0.1\gamma_{e-h}$ where $\overline{N}_e$ is average densities of electrons). Trion term yields better agreement at the delays around $\Delta t \approx -5$ ps (solid red line in Fig. S8). However, higher order terms exhibit a much steeper dependence on fluence compared to the experimental observations, especially at high fluence (solid blue line in Fig. S8). Considering a marginal improvement of simulations incorporating additional higher order terms at higher fluence and to maintain model simplicity, we have limited discussion of higher order terms in the main text.

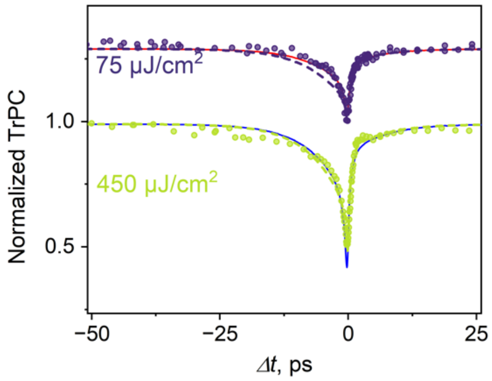


**Fig S8. Fluence dependence of interlayer trion contributions to trPC.** Simulated dynamics of TrPC including interlayer excitons and interlayer trion contributions. To model trion dynamics we use additional non-linear term $-\gamma_T N_e^2 N_h$ in Eq. 1 of the main text. The parameter values yielding the best fit were found to be $\gamma_{e-h} = 0.085$ cm$^2$/s; $\gamma_T = 8{\cdot}10^{17}$ cm$^4$/s and $\tau_e, \tau_h, m$ kept the same as in the main text (solid red and blue lines). Compared to exciton-only case used in the main text (dashed purple and green line), decay at $\Delta t \approx -5$ ps is captured better. However, the inclusion of trions in the model leads to a worse fit at higher fluences.

## Supplementary Note 2: Methods.

*Fabrication*: The $MoSe_2$ and $MoS_2$ monolayers were obtained by mechanical exfoliation of bulk crystals (>99.9% pure synthetic crystals from HQ graphene) onto PDMS. Then the $MoS_2$/$MoSe_2$ heterostructure was built on PDMS via direct pick-up[11]. The twist angle is estimated using the edges of the flakes. The two flakes are intentionally misaligned (>3°) so that the excitonic ground state is dark. Next, the heterostructure was transferred onto an hBN flake (Scotch tape exfoliated) on Si/$SiO_2$ (295 nm) substrate. The samples were washed with acetone/IPA and later annealed in a vacuum at 230 °C for ~12 h to remove the organic residues. The electrical connections were defined using electron beam lithography (Raith Pioneer II) at low exposure (10 µC/cm$^2$) to prevent contamination [12]. Finally, 95 nm of Au with an adhesion layer of 5 nm of Cr was thermally evaporated on top, followed by lift-off. In total, three samples were produced and measured. The results presented in the main text are from sample D1 (The results from other samples are in the Supplementary Information, Fig. S10).

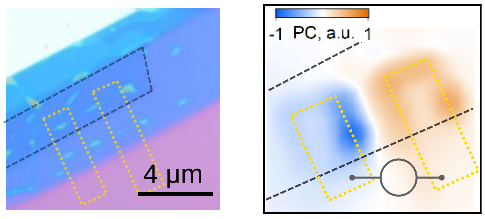


**Fig. S9** Optical image of typical device (left). Static photocurrent map measured at $MoSe_2$ resonance (right). Photocurrent gives high response in the heterostructure region (grey dashed line) and is greatly amplified near electrodes (yellow dashed rectangles).

*Static PC spectroscopy:* we use optical pulses generated by an ultrafast laser system (tunable Ti:Sa oscillator and optical parametric oscillator, pulse width ~150 fs) that are focused into a diffraction-limited

spot onto a sample kept in vacuum (~$10^{-5}$ mbar) at room temperature (Fig. S9). No bias voltage is applied across the sample. In spatial scans across the electrically unbiased heterostructure, the PC is maximum within the areas near the electrodes (Fig. S9), where photoexcited carriers are converted into the current with high efficiency.

*Pump-probe spectroscopy:* The samples were measured in a cryostat under high vacuum ($10^{-5}$ mbar). The cryostat was mounted on a motor-controlled xy-translation stage with sub-micrometer spatial resolution. We used a wavelength-tunable femtosecond pulsed laser system (Coherent Chameleon Ultra II + compact OPO-VIS) with pulse duration of ~150 fs and 80 MHz repetition rate. The pulses are time-delayed with ~10 fs precision using an optical delay line. Both pulses were focused into a diffraction-limited spot. The pulse fluence is varied using a combination of a λ/2 wave plate and a cube polarizer. The photocurrent signal was measured using a lock-in amplifier (SR 830) phase-locked to a chopper in the optical path. For the trRef measurement, we use the same lock-in technique with a chopper in the pump path, and only the probe beam is sent to a photodetector (Thorlabs PDB450A).

*Optical measurements:* The PL data (Fig. 2b of the main text) were acquired with an XploRA™ HORIBA using 532 nm excitation at 16 µW power focused into a diffraction-limited spot (≈1 µm diameter).

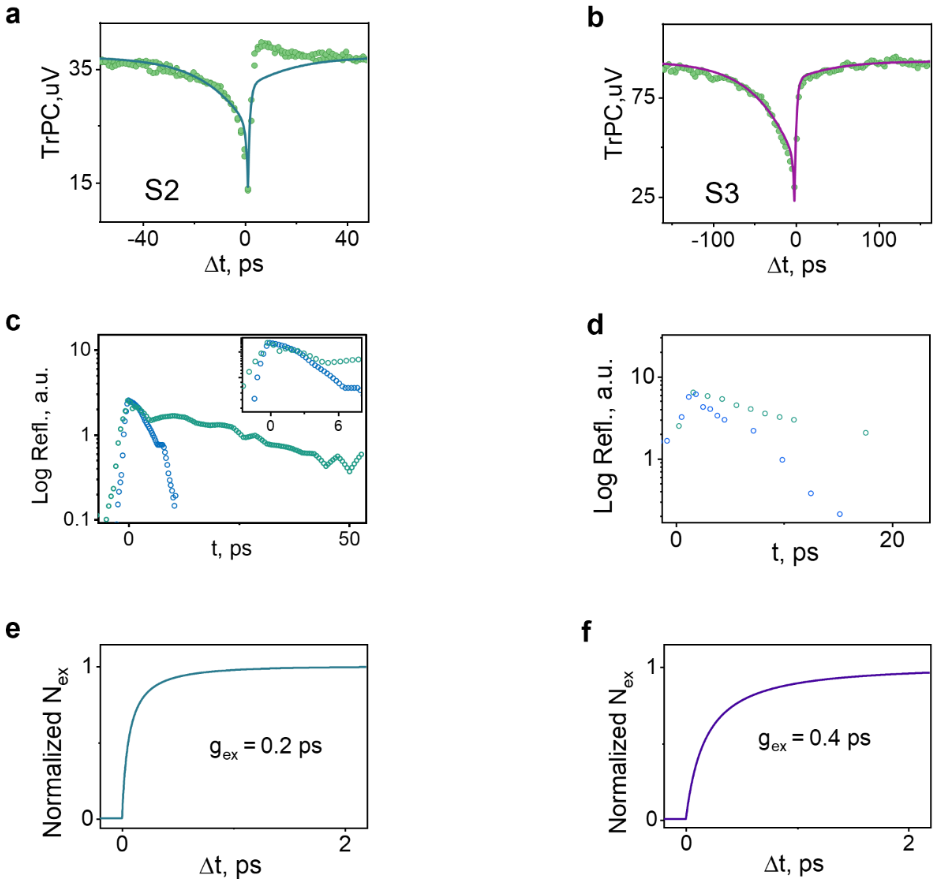


**Fig S10. trPC and trRef response of different samples**. **a-b)** TrPC response of additional samples S2, S3 (points) along with the results of simulations using Eq. 1 of the main text using best-fit parameters (solid lines). For S2 the fit parameters are: $\tau_e$ =1.6 ps, $\tau_h$ = 15.2 ps, e-h interaction strength $\gamma_{e-h}$ =0.25 cm²/s electron/hole tunneling efficiency $m$ = 75 % . For S3, we find: $\tau_e$ =2.4 ps, $\tau_h$ = 41.0 ps, $\gamma_{e-h}$ =0.2 cm²/s, $m$ = 55 %. We see similar trends (asymmetry of trPC for positive and negative time, fast electron decay and slow hole decay, close values of interaction strengths and tunneling efficiency) as in the device S1 shown in the main text. One notable difference compared to S1, a small increase of photocurrent at around $\Delta t$ = 5 ps in (a) could be related to photodoping-induced increase in hole extraction efficiency. **c-d)** Transient reflectivity dynamics (trRef) of S2, S3 with the $MoS_2$ ($MoSe_2$) bandgap resonant pump and the $MoSe_2$ ($MoS_2$) bandgap resonant probe in green (blue) points. The decay of both charge carriers is coupled in first ~3 ps, while holes have almost order of magnitude longer lifetime. **e-f)** The density of interlayer exciton in samples S2 and S3 obtained from best fit parameters. Variation in the parameters of different heterostructures is likely related to different twist angle.

**Supplementary Note 3: Two-color time-resolved reflectivity.**

To independently from trPC check the dynamics of free carriers in the $MoS_2/MoSe_2$ heterostructure, we carry out a time-resolved reflectivity measurements[13]. In this approach, one optical pulse, e.g., in resonance with the $MoSe_2$ bandgap, excites holes and electrons. Part of the electron population is transferred to the CBM in $MoS_2$. These indirectly excited electrons change the reflectivity of $MoS_2$, probed by the second pulse in resonance with the $MoS_2$ bandgap. This optical measurement provides access to the dynamics of transferred electrons. While ongoing processes are the same as in trPC measurement, trRef measures the dynamics of transferred electrons, and trPC is sensitive to the dynamics of holes excited by the first pulse. Conversely, pumping at $MoS_2$ and probing at $MoSe_2$ resonances is sensitive to dynamics of indirectly excited holes. We observe that the indirectly excited electrons decay fast with a time constant similar to what is observed in photocurrent measurements (blue in Fig. S11). In contrast, indirectly excited holes (green in Fig. S11) first decay with a rate similar to that of electrons ($\Delta t < 1$ ps) while for larger delays ($\Delta t \gg 1$ ps) the decay slows down to the rate similar to that measured via trPC for holes (inset in Fig. S11). To quantitatively extract $\tau_{e/h}$ from optical measurements and compare them with those from photocurrent, we assume that strength of the reflectivity signal is proportional to density of free carriers. For holes, for example, the solution of Eq. 1 with $m \neq 0$ is (Supplementary note 5)

$$trRef(\Delta t) \sim m\, e^{-\frac{\Delta t}{\tau_h}} \cdot e^{-\gamma N_e^0 \tau_e \left(1 - e^{-\frac{\Delta t}{\tau_e}}\right)}. \quad (1)$$

The first exponent describes the linear decay of holes in a non-interacting case, while the second exponent corresponds to the nonlinear acceleration of the decay due to electron-hole coupling. Since $\tau_e \ll \tau_h$, the reflectivity signal is dominated by the second part for small delays, $trRef(\Delta t \to 0) \sim e^{-\gamma N_e^0 \tau_e \Delta t}$. After the decay of the electron population, the hole dynamics are governed by the first term, $trRef(\Delta t \gg \tau_e) \sim e^{-\Delta t/\tau_h}$. Using a version of Eq. 1 for electrons and holes, we fit the reflectivity signal for the cases of $MoS_2$ and $MoSe_2$ probing (solid green and blue lines in Fig. S11). The resulting time constants of electrons and holes, $\tau_e = 1.4$ ps and $\tau_h = 6.3$ ps, are close to what is obtained from trPC. In this model, the formation of dark excitons is the reason behind the bi-exponential decay observed here (Fig. S11) and in other works[2,13]. Note, that the trRef signal also depends on the exciton formation rate $\gamma_{e-h}$. Unlike the trPC signal, for which the entire signal is proportional to $\gamma_{e-h}$ (Eq. 2 of the main text), in the trRef case, $\gamma_{e-h}$ only modifies the decay shortly after the excitation. Moreover, other effects such as carrier cooling and band renormalization may also influence the decay of trRef at short timescales [14,15].

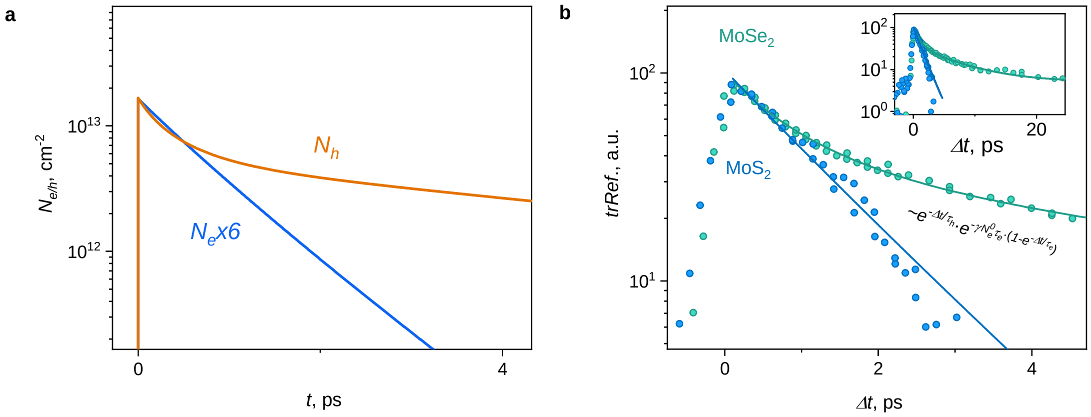


**Fig. S11 Simulated dynamics of charge carriers and trRef response. a)** Semilog plot of simulated dynamics of electrons and holes tunneled after $P_{MoSe2}$, $P_{MoS2}$ excitation, respectively, those are probed in idealized trRef measurement. Decay of electrons and holes up to ~2 ps after excitation is the same as it is dominated by $\gamma_{e-h}$ (Eq. 1). **b)** Semilog plot of time-resolved reflectivity signal of the $MoS_2/MoSe_2$ heterostructure. Green points correspond to the probe pulse in resonance with $MoSe_2$ bandgap (pump $MoS_2$), blue – in resonance with $MoS_2$ bandgap (pump $MoSe_2$). The inset shows a longer timescale. Lines are fits (Eq. 1) derived from the model in Eq. 1 of the main text.

**Supplementary Note 4: Analytical solution of the charge carriers and excitons dynamics and resulting photocurrent.**

Our goal is to get an approximate solution of nonlinear equation 1 of the main text describing the dynamics of charge carriers and calculate the resulting time-resolved photocurrent response (trPC). First, we assume zero carrier transfer efficiency ($m = 0$). In this case, the first pulse at time $t = -\Delta t$ generates a density of holes $N_h^0$ and the second pulse, at zero time, excites electron density $N_e^0$. Next, we solve it in the limit of electron-hole interaction $\left(\gamma_{e-h} \coloneqq \gamma \ll \frac{1}{N_{e/h}\tau_{e/h}}\right)$, and slower decay rate of holes than electrons ($\tau_h \gg \tau_e$). The problem can be summarized as follows:

$$\begin{cases} \dfrac{dN_e}{dt} = -\dfrac{N_e}{\tau_e} - \gamma N_e N_h \\ \dfrac{dN_h}{dt} = -\dfrac{N_h}{\tau_h} - \gamma N_e N_h \\ \dfrac{dN_{ex}}{dt} = \gamma N_e N_h \\ N_e(0) = N_e^0;\ N_h(-\Delta t) = N_h^0;\ N_{ex}(0) = 0 \end{cases} \tag{2}$$

As discussed in the Supplementary Note 1, hole contribution to the photocurrent is much larger due to the higher extraction efficiency and longer lifetime. Therefore, we analyze only the contribution of holes to trPC. As mentioned in the main text, the time-resolved photocurrent measured in a lock-in measurement can be expressed as

$$TrPC(\Delta t) \sim \int_{-\infty}^{+\infty} N_h(t, \mathrm{N_e^0} = 0)dt - \int_{-\infty}^{+\infty} N_h(t, \Delta t)dt \tag{3}$$

First, we solve differential equations in the simplest case $\gamma = 0$. The system (2) is then reduced to

$$\begin{cases} \dfrac{dN_e}{dt} = -\dfrac{N_e}{\tau_e} \\ \dfrac{dN_h}{dt} = -\dfrac{N_h}{\tau_h} \\ \dfrac{dN_{ex}}{dt} = \gamma_{e-h} N_e N_h \\ N_e(0) = N_e^0;\ N_h(-\Delta t) = N_h^0;\ N_{ex}(0) = 0 \end{cases}$$

We find the following solution:

$$\begin{cases} N_e = A \cdot e^{-t/\tau_e} \\ N_h = B \cdot e^{-t/\tau_h} \end{cases} \tag{4}$$

the constants are defined by the boundary conditions: $A = N_e^0$ and $B = N_h^0 \cdot e^{-\Delta t/\tau_h} := N_h^*$, where we introduced $N_h^*$ - density of holes excited by the first pulse and decayed after $\Delta t$. In this case, the $N_h$ is independent of $N_e$ therefore integrals in Eq. 3 are equal and trPC is zero.

To solve the system in the first order of $\gamma \ll \frac{1}{N_{e/h}\tau_{e/h}}$ , we treat Eq. 4 as an ansatz with time-dependent constants $A(t)$ and $B(t)$ and we neglect all terms which scale as $\gamma^2$. We plug (4) into (2):

$$\begin{cases} \frac{dA}{dt} \cdot e^{-\frac{t}{\tau_e}} - A \cdot \frac{e^{-t/\tau_e}}{\tau_e} = -\frac{A \cdot e^{-t/\tau_e}}{\tau_e} - \gamma A \cdot e^{-t/\tau_e} B \cdot e^{-t/\tau_h} \\ \frac{dB}{dt} \cdot e^{-\frac{t}{\tau_h}} - B \cdot \frac{e^{-t/\tau_h}}{\tau_h} = -\frac{B \cdot e^{-t/\tau_h}}{\tau_h} - \gamma A \cdot e^{-t/\tau_e} B \cdot e^{-t/\tau_h} \end{cases} \tag{5}$$

We simplify the system (5) and replace $A, B$ in the first order of $\gamma$ by constants $A_0$ and $B_0$ in the right part of (5):

$$\begin{cases} \dfrac{dA}{dt} = -\gamma A B_0 \cdot e^{-t/\tau_h} \\ \dfrac{dB}{dt} = -\gamma A_0 B \cdot e^{-t/\tau_e} \end{cases}$$

We get:

$$\begin{cases} A = C_0 \exp\left(\gamma B_0 \tau_h e^{-\frac{t}{\tau_h}}\right) \\ B = D_0 \exp\left(\gamma A_0 \tau_e e^{-\frac{t}{\tau_e}}\right) \end{cases}$$

To find the constants we again use the boundary conditions:

$$\begin{cases} A(0) = C_0 \exp(\gamma B_0 \tau_h) = N_e^0 \\ B(0) = D_0 \exp(\gamma A_0 \tau_e) = N_h^* \end{cases} \leftrightarrow \begin{cases} C_0 = N_e^0 \exp(-\gamma B_0 \tau_h) \approx N_e^0 (1 - \gamma B_0 \tau_h) \approx N_e^0 (1 - \gamma N_h^* \tau_h) \\ D_0 = N_h^* \exp(-\gamma A_0 \tau_e) \approx N_h^* (1 - \gamma A_0 \tau_e) \approx N_h^* (1 - \gamma N_e^0 \tau_e) \end{cases}$$

Here we used the expansion for $\gamma N_{e/h} \tau_{e/h} \to 0$. Then the solution of the system (2) in the first order of $\gamma$ for positive time is given by:

$$\begin{cases} N_e = N_e^0 \exp\left(-\dfrac{t}{\tau_e} - \gamma N_h^* \tau_h \left(1 - e^{-\frac{t}{\tau_h}}\right)\right) \\ N_h = N_h^* \exp\left(-\dfrac{t}{\tau_h} - \gamma N_e^0 \tau_e \left(1 - e^{-\frac{t}{\tau_e}}\right)\right) \end{cases}$$

The full solution in the entire time domain is given by:

$$N_h = \begin{cases} N_h^0 e^{-\frac{t+\Delta t}{\tau_h}}, & -\Delta t \le t < 0 \\ N_h^0 \exp\left(-\dfrac{t+\Delta t}{\tau_h} - \gamma N_e^0 \tau_e \left(1 - e^{-\frac{t}{\tau_e}}\right)\right), & 0 \le t \end{cases} \quad (6)$$

To find trPC, we need to integrate $N_h$. The integral is separated into two for both ranges of $t$. The first one is simple, $\int_{-\Delta t}^{0} N_h(t, \Delta t) dt = N_h^0 \tau_h (1 - e^{-\Delta t/\tau_h})$. The integral over the second range is given by Γ-functions and can be further simplified using properties of Γ – function, and expansion at $\gamma \tau_h N_e \to 0$:

$$\int_0^{+\infty} N_h(t, \Delta t) dt = N_h^0 \cdot e^{-\frac{\Delta t}{\tau_h}} \cdot e^{-\gamma \tau_e N_e^0} \left(\tau_h + \frac{\tau_h \tau_e^2}{\tau_e + \tau_h} \gamma N_e^0\right) \quad (7)$$

$$\to \int_{-\infty}^{+\infty} N_h(t, \Delta t) dt = N_h^0 \tau_h \cdot \left(1 + e^{-\frac{\Delta t}{\tau_h}} \cdot \left[e^{-\gamma \tau_e N_e^0} \left(1 + \frac{\tau_e^2}{\tau_e + \tau_h} \gamma N_e^0\right) - 1\right]\right)$$

Using Eq. 3 we derive the trPC:

$$TrPC(\Delta t) \sim -N_h^0 \tau_h e^{-\frac{\Delta t}{\tau_h}} \cdot \left[e^{-\gamma \tau_e N_e^0} \left(1 + \frac{\tau_e^2}{\tau_e + \tau_h} \gamma N_e^0\right) - 1\right] \approx \left| \begin{matrix} \gamma \tau_e N_e^0 \ll 1 \\ e^{-\gamma \tau_e N_e^0} \approx (1 - \gamma \tau_e N_e^0) \end{matrix} \right| \approx$$

$$\approx -N_h^0 \tau_h e^{-\frac{\Delta t}{\tau_h}} \cdot \left[(1 - \gamma \tau_e N_e^0) \left(1 + \frac{\tau_e^2}{\tau_e + \tau_h} \gamma N_e^0\right) - 1\right]$$

$$\approx -N_h^0 \tau_h e^{-\frac{\Delta t}{\tau_h}} \cdot \left[\frac{\tau_e^2}{\tau_e + \tau_h} \gamma N_e^0 - \gamma \tau_e N_e^0\right] = N_h^0 N_e^0 \gamma \tau_h e^{-\frac{\Delta t}{\tau_h}} \cdot \frac{\tau_e \tau_h}{\tau_e + \tau_h} \approx \left| \begin{matrix} \tau_e \ll \tau_h \\ \dfrac{\tau_e \tau_h}{\tau_e + \tau_h} \approx \tau_e \end{matrix} \right|$$

$$\approx N_h^0 N_e^0 \gamma \tau_h \tau_e e^{-\frac{\Delta t}{\tau_h}}$$

Note that trPC depends on the density of holes which have not decayed until the second, electron exciting, pulse ($N_h^0 e^{-\Delta t/\tau_h} = N_h^*$): $TrPC(\Delta t) \sim N_h^0 N_e^0 \gamma \tau_h \tau_e e^{-\Delta t/\tau_h} = N_h^* N_e^0 \gamma \tau_h \tau_e$. When electrons are excited first and after $\Delta t$ holes, the trPC similarly depends on the density of survived electrons:

$TrPC(\Delta t) \sim N_h^0 N_e^0 \gamma \tau_h \tau_e e^{-\frac{\Delta t}{\tau_e}}$. Both cases combined yield the final formula used in the main text:

$$TrPC(\Delta t) \sim N_h^0 N_e^0 \gamma \tau_h \tau_e e^{-\frac{\Delta t}{\tau_{h/e}}} \tag{8}$$

**Supplementary Note 5 Dynamics in case of non-zero transfer efficiency ($m \neq 0$).**

Our goal is to show how indirectly excited holes (holes that need to transfer to the VBM of the heterostructure) influence trPC response. For that, we solve Eq. 1 with the pulse exciting predominantly electron first and holes - second, which corresponds to $\Delta t > 0$ in Fig. 1 of the main text. As explained in the previous note, trPC depends on the density of carriers after the first pulse that persists when the second pulse arrives ($N_e^*$ and $N_h^*$). Therefore, we just need to find the influence of $m$ on $N_e^*$ and $N_h^*$. Similar to the previous note, we assume small non-linearity ($\gamma \ll \frac{1}{N_{e/h}\tau_{e/h}}$), and slower decay rate of holes compared to electrons ($\tau_h \gg \tau_e$). Unlike the previous note, we now solve Eq.1 for the case of non-zero $m$, and large $\Delta t$. In this limit, only holes are left.

The decay of holes and electrons after excitation of both carriers in presence of small nonlinearity is described by the second part of Eq. 6, where we assume that in $MoS_2$ equal number of electrons ($N_e^0$) and holes ($N_h^{\mathrm{MoS2}}$) is excited and $m$ part is transferred $N_h^0 = mN_h^{\mathrm{MoS2}} = mN_e^0$:

$$\begin{cases} N_e = N_e^0 \exp\left(-\frac{t}{\tau_e} - \gamma m N_e^0 \tau_h \left(1 - e^{-\frac{t}{\tau_h}}\right)\right) \\ N_h = mN_e^0 \exp\left(-\frac{t}{\tau_h} - \gamma N_e^0 \tau_e \left(1 - e^{-\frac{t}{\tau_e}}\right)\right) \end{cases}$$

The second part of the system describes the dynamics of holes that transferred to VBM maximum ($MoSe_2$) after the excitation of electrons directly in CBM ($MoS_2$) with an optical pulse, which is measured in trRef. Assuming that reflectivity is proportional to the density of carriers and fluence of the probe pulse, then the time-resolved response can be naively described by:

$$\mathrm{trRef} \sim m P_{\mathrm{MoSe2}} P_{\mathrm{MoS2}} \exp\left(-\frac{\Delta t}{\tau_h} - \frac{\gamma P_{\mathrm{MoS2}} f A}{S} \tau_e \left(1 - e^{-\frac{\Delta t}{\tau_e}}\right)\right) \tag{9}$$

Where $A$ is the absorption of the flake, $f$ the repetition rate of the laser, $S$ is – the area of the beam spot.

The second pulse at $t = \Delta t \gg \tau_e$ co-excite $mN_h^0$ electrons through the interlayer transfer mechanism, while elections from the first pulse already decayed as $\tau_e \ll \tau_h$. The density of holes is given by the sum of holes excited by the second pulse and residual holes left after the first pulse.

$$\begin{cases} N_e(0) = mN_h^0 \\ N_h(0) = N_h^0 + mN_e^0 \exp\left(-\frac{\Delta t}{\tau_h} - \gamma N_e^0 \tau_e \left(1 - e^{-\frac{\Delta t}{\tau_e}}\right)\right) \end{cases} \tag{10}$$

Then the dynamics of holes after the second pulse are described by:

$$N_h(t) = \left[N_h^0 + mN_e^0 \exp\left(-\frac{\Delta t}{\tau_h} - \gamma N_e^0 \tau_e \left(1 - e^{-\frac{\Delta t}{\tau_e}}\right)\right)\right] \cdot \exp\left(-\frac{\Delta t}{\tau_h} - \gamma m N_h^0 \tau_e \left(1 - e^{-\frac{\Delta t}{\tau_e}}\right)\right)$$

To calculate trPC we use Eq. 3 which consists of two integrals: two pulse excitation and only pulse that excites holes. The second integral is given by:

$$\int_{-\infty}^{+\infty} N_h(t, \mathrm{N_e^0} = 0) dt = \int_{-\infty}^{+\infty} N_h^0 \cdot \exp\left(-\frac{\Delta t}{\tau_h} - \gamma m N_h^0 \tau_e \left(1 - e^{-\frac{\Delta t}{\tau_e}}\right)\right) dt$$

The integral is similar to Eq. 7 where $N_e^0 = mN_h^0$ and $\Delta t = 0$:

$$\int_{-\infty}^{+\infty} N_h(t, \mathrm{N}_\mathrm{e}^0 = 0)dt = N_h^0 \cdot e^{-\gamma\tau_e m N_h^0}\left(\tau_h + \frac{\tau_h\tau_e^2}{\tau_e+\tau_h}\gamma m N_h^0\right) \tag{11}$$

Since m≠ 0, the integral for two pulse trPC breaks into two time ranges from $-\Delta t$ to 0 and from 0 to $+\infty$. The first integral is essentially the same as Eq. 8 but the limited range makes it harder to calculate, therefore, we use $\gamma = 0$ solution (Eq. 3) instead of a more accurate Eq. 10. The integral over the second range is given by a formula similar to Eq. 11, but the initial density is replaced by Eq. 10:

$$\begin{aligned}\int_{-\infty}^{+\infty} N_h(t, \Delta\mathrm{t})dt &= \int_{-\Delta t}^{0} N_h(t, \Delta\mathrm{t})dt + \int_{0}^{+\infty} N_h(t, \Delta\mathrm{t})dt \\ &= mN_e^0\tau_h\left(1 - e^{-\frac{\Delta t}{\tau_h}}\right) \\ &+ \left(N_h^0 + mN_e^0 \exp\left(-\frac{\Delta t}{\tau_h} - \gamma N_e^0\tau_e\left(1 - e^{-\frac{\Delta t}{\tau_e}}\right)\right)\right)e^{-\gamma\tau_e m N_h^0}\left(\tau_h + \frac{\tau_h\tau_e^2}{\tau_e+\tau_h}\gamma m N_h^0\right)\end{aligned}$$

Using Eq. 2 we get trPC:

$$trPC(\Delta t) \approx -mN_e^0\tau_h\left(1 - \gamma\tau_e e^{-\frac{\Delta t}{\tau_h}}(N_e^0 + mN_h^0)\right) \approx mN_e^0\tau_h\gamma\tau_e e^{-\frac{\Delta t}{\tau_h}}(N_e^0 + mN_h^0)$$

Where we removed the constant as it does not depend on $\Delta t$. The $N_e^0$ in the brackets is a single pulse response which appears due to simplification ($\gamma = 0$) for $-\Delta t; 0$ range. If we leave only terms relevant for two pulses response:

$$trPC(\Delta t) \sim m^2 N_e^0 N_h^0 \gamma\tau_h\tau_e e^{-\frac{\Delta t}{\tau_h}} \tag{12}$$

We demonstrated that the decay at large delay time in the case of non-zero transfer is defined by $\tau_h$, while at small delay times it is $\tau_e$ as we have shown before (see Supplementary Note 4). Moreover, the amplitude of this component is proportional to $m^2$.


1. Massicotte, M. *et al.* Dissociation of two-dimensional excitons in monolayer $WSe_2$. *Nat Commun* **9**, 1633 (2018).

2. Ceballos, F., Bellus, M. Z., Chiu, H.-Y. & Zhao, H. Ultrafast Charge Separation and Indirect Exciton Formation in a $MoS_2$ –$MoSe_2$ van der Waals Heterostructure. *ACS Nano* **8**, 12717–12724 (2014).

3. Sun, D. *et al.* Observation of Rapid Exciton–Exciton Annihilation in Monolayer Molybdenum Disulfide. *Nano Lett* **14**, 5625–5629 (2014).

4. Zhu, H. *et al.* Interfacial Charge Transfer Circumventing Momentum Mismatch at Two-Dimensional van der Waals Heterojunctions. *Nano Lett* **17**, 3591–3598 (2017).

5. Choi, C. *et al.* Enhanced interlayer neutral excitons and trions in trilayer van der Waals heterostructures. *NPJ 2D Mater Appl* **2**, (2018).

6. Miller, B. *et al.* Long-Lived Direct and Indirect Interlayer Excitons in van der Waals Heterostructures. *Nano Lett* **17**, 5229–5237 (2017).

7. Wang, J. *et al.* Optical generation of high carrier densities in 2D semiconductor heterobilayers. *Sci Adv* **5**, (2019).

8. Wang, J. *et al.* Diffusivity Reveals Three Distinct Phases of Interlayer Excitons in MoSe 2 =WSe 2 Heterobilayers. (2021) doi:10.1103/PhysRevLett.126.106804.

9. Yuan, L. *et al.* Twist-angle-dependent interlayer exciton diffusion in WS2–WSe2 heterobilayers. *Nat Mater* **19**, 617–623 (2020).

10. Tanoh, A. O. A. *et al.* Directed Energy Transfer from Monolayer WS $_2$ to Near-Infrared Emitting PbS–CdS Quantum Dots. *ACS Nano* **14**, 15374–15384 (2020).

11. Baek, H. *et al.* Highly energy-tunable quantum light from moiré-trapped excitons. *Sci Adv* **6**, 8526–8537 (2020).

12. Yagodkin, D. *et al.* Extrinsic Localized Excitons in Patterned 2D Semiconductors. *Adv Funct Mater* **32**, 2203060 (2022).

13. Kumar, A. *et al.* Spin/Valley Coupled Dynamics of Electrons and Holes at the $MoS_2$ – $MoSe_2$ Interface. *Nano Lett* **21**, 7123–7130 (2021).

14. Chernikov, A., Ruppert, C., Hill, H. M., Rigosi, A. F. & Heinz, T. F. Population inversion and giant bandgap renormalization in atomically thin $WS_2$ layers. *Nature Photonics 2015 9:7* **9**, 466–470 (2015).

15. Nie, Z. *et al.* Ultrafast Carrier Thermalization and Cooling Dynamics in Few-Layer $MoS_2$. *ACS Nano* **8**, 10931–10940 (2014).
